# Supplementary material for: Proton-triggered rearrangement of the AMPA receptor N-terminal domains impacts receptor kinetics and synaptic localization
Source: Nat Struct Mol Biol. 2024 Aug 13;31(10):1601–13. doi: 10.1038/s41594-024-01369-5 (PMC11479944; doi:10.1038/s41594-024-01369-5)
Supplement: Supplementary file 1 — Reporting Summary [file 41594_2024_1369_MOESM1_ESM.pdf]

nature portfolio

Corresponding author(s): Ingo Greger, Terunaga Nakagawa

Last updated by author(s): Jun 17, 2024

Reporting Summary

Nature Portfolio wishes to improve the reproducibility of the work that we publish. This form provides structure for consistency and transparency in reporting. For further information on Nature Portfolio policies, see our Editorial Policies and the Editorial Policy Checklist.

Statistics

For all statistical analyses, confirm that the following items are present in the figure legend, table legend, main text, or Methods section.

n/a	Confirmed	
☐	☒	The exact sample size (*n*) for each experimental group/condition, given as a discrete number and unit of measurement
☐	☒	A statement on whether measurements were taken from distinct samples or whether the same sample was measured repeatedly
☐	☒	The statistical test(s) used AND whether they are one- or two-sided<br>*Only common tests should be described solely by name; describe more complex techniques in the Methods section.*
☒	☐	A description of all covariates tested
☐	☒	A description of any assumptions or corrections, such as tests of normality and adjustment for multiple comparisons
☐	☒	A full description of the statistical parameters including central tendency (e.g. means) or other basic estimates (e.g. regression coefficient) AND variation (e.g. standard deviation) or associated estimates of uncertainty (e.g. confidence intervals)
☐	☒	For null hypothesis testing, the test statistic (e.g. *F*, *t*, *r*) with confidence intervals, effect sizes, degrees of freedom and *P* value noted<br>*Give P values as exact values whenever suitable.*
☒	☐	For Bayesian analysis, information on the choice of priors and Markov chain Monte Carlo settings
☒	☐	For hierarchical and complex designs, identification of the appropriate level for tests and full reporting of outcomes
☒	☐	Estimates of effect sizes (e.g. Cohen's *d*, Pearson's *r*), indicating how they were calculated

Our web collection on statistics for biologists contains articles on many of the points above.

Software and code

Policy information about availability of computer code

Data collection	EEPU v2.6-3.3 (Thermofisher Scientific), pClamp10 (Molecular Devices)
Data analysis	Relion v4 and v5 (open source), CTFFIND4 (open source), motioncorr2 (open source), Resmap v1.95 (open source), coot v0.897 (for OSX, open source), chimera v1.14 (open source) , pymol v2.1 (Schrodinger), phenix v1.20 (open source), AlphaFold2, DynaMut2,Microsoft Excel v16 (Microsoft), Prism v10 (Graph Pad Software).

For manuscripts utilizing custom algorithms or software that are central to the research but not yet described in published literature, software must be made available to editors and reviewers. We strongly encourage code deposition in a community repository (e.g. GitHub). See the Nature Portfolio guidelines for submitting code & software for further information.

Data

Policy information about availability of data

All manuscripts must include a data availability statement. This statement should provide the following information, where applicable:

- Accession codes, unique identifiers, or web links for publicly available datasets
- A description of any restrictions on data availability
- For clinical datasets or third party data, please ensure that the statement adheres to our policy

Cryo-EM coordinates and corresponding EM-maps are deposited in the PDB and EMDB under the following accession codes; PDB:9B5Z (EMD-44232), PDB:9B60 (EMD-44233), PDB:9B67 (EMD-44248), PDB:9B68 (EMD-44249), PDB:9B6A (EMD-44251), PDB:9B69 (EMD-44250), PDB:9B61 (EMD-44234), PDB:9B63 (EMD-

44244), and PDB:9B64 (EMD-44245). Molecular dynamic (MD) simulation trajectories are deposited in Zenodo with the following DOI: 10.5281/zenodo.11654387. Request for materials (plasmids and cell lines) will be fulfilled for reasonable inquiries and should be addressed to Ingo Greger and Terunaga Nakagawa. Source data are provided with this paper.

Human research participants

Policy information about studies involving human research participants and Sex and Gender in Research.

Reporting on sex and gender	n/a
Population characteristics	n/a
Recruitment	n/a
Ethics oversight	n/a

Note that full information on the approval of the study protocol must also be provided in the manuscript.

Field-specific reporting

Please select the one below that is the best fit for your research. If you are not sure, read the appropriate sections before making your selection.

☒ Life sciences ☐ Behavioural & social sciences ☐ Ecological, evolutionary & environmental sciences

For a reference copy of the document with all sections, see nature.com/documents/nr-reporting-summary-flat.pdf

Life sciences study design

All studies must disclose on these points even when the disclosure is negative.

Sample size	Sample size for cryo-EM data collection was determined based on the knowledge that AMPARs require about 20-100 thousand particles to reach 3.5Å resolution. To to be able to sort out conformational variability of the NTDs by classifying itno 20-40 classes we estimated that about 1,000,000 ± 200,000 particles are needed, which translates into collecting about 20,000 micrographs (Zhang, Nature 2023, Nakagawa, NSMB 2024).<br>Electrophysiology sample sizes were determined based on literature review, previous experience with data of this sort, and reproducibility of results across independent experiments. The authors have extensive previous experience with data of this type (Zhang, Nature 2023&2021; Herguedas, Science 2019; Herguedas, Science 2016; Cais, Cell Reports 2014)., therefore sample sizes were based on understanding of sample variabilities.<br>Light microscopy sample sizes were determined based on previous FRAP experiments (Watson, eLife 2017) and were reproducible across recordings from multiple cells from three different culture preparations.
Data exclusions	CTF and rlnMaxResolution parameters were used to remove images with bad image quality. 3D classification removes particles based on objective statistical measures which retains particles with homogeneous structures containing high resolution signals. FRAP recordings were excluded or included based on cell viability and stability of conditions during recording.
Replication	Expression and purification were highly robust and reproducible across experiments. The half maps of the 3D refinement in each structure produced consistent results, which supports high consistency of data quality across micrographs. All electrophysiology data sets were pooled from at least two independent experiments and all results were successfully replicated at least five times. Light microscopy experiments were replicated from multiple cells across three different culture preparations.
Randomization	For Cryo-EM, division of datasets into two random halves was done based on standard approach in RELION. Randomization is not relevant to electrophysiology. Similarly for imaging experiments, the experimenter is in charge of handling plasmids, cell lines, transfection and acquiring the data on the microscope so it is not feasible to randomise.
Blinding	Blinding was not applicable to cryo-EM or MD simulations, because this type of study does not use group allocation. Researchers were not blinded for the acquisition or analysis of electrophysiology and imaging data as it was not technically or practically feasible to do so. Experimenter independence was ensured by application of defined exclusion criteria as stated above.

Reporting for specific materials, systems and methods

We require information from authors about some types of materials, experimental systems and methods used in many studies. Here, indicate whether each material, system or method listed is relevant to your study. If you are not sure if a list item applies to your research, read the appropriate section before selecting a response.

Materials & experimental systems

n/a	Involved in the study	
☐	☒	Antibodies
☐	☒	Eukaryotic cell lines
☒	☐	Palaeontology and archaeology
☐	☒	Animals and other organisms
☒	☐	Clinical data
☒	☐	Dual use research of concern

Methods

n/a	Involved in the study	
☒	☐	ChIP-seq
☒	☐	Flow cytometry
☒	☐	MRI-based neuroimaging

Antibodies

Antibodies used	anti-FLAG M2 monoclonal antibody
Validation	Purchased from Sigma. The product is quality controlled.

Eukaryotic cell lines

Policy information about cell lines and Sex and Gender in Research

Cell line source(s)	HEK293Tcells were purchased from ATCC and TetON HEK cell (Clontech) and their derivatives were isolated in Nakagawa lab.
Authentication	No further authentication of HEK293T was performed for cell lines used in the electrophysiology experiments. The TetON HEK cell line was purchased from Clontech. The cell morphology is spindle shaped and homogeneous. Growth rate was consistent with HEK cell. The cell line respond to DOX as described by the manufacturer. The cell line is sensitive to hygromycine and zeocin. The cell line is insensitive to G418. The line is used extensively in past literatures to generate stable cell lines that DOX dependently express proteins for structural studies.
Mycoplasma contamination	No mycoplasma testing was performed specifically for this study, the HEK293T cell line had been tested negative in the past.
Commonly misidentified lines<br>(See ICLAC register)	HEK cells are listed in the register; however, our HEK cell lines come from reliable source (ATCC) and are the only secondary cell type used in this study, which minimizes the risk of any cross-contamination.

Animals and other research organisms

Policy information about studies involving animals; ARRIVE guidelines recommended for reporting animal research, and Sex and Gender in Research

Laboratory animals	C57/Bl6 mice of both sexes were used in this study at age postnatal day 0-1. Animals were housed with unlimited access to food and water under a standard 12 hour light-dark cycle, at normal room temperature (approx 20-22 degrees Centigrade). Pregnant mothers were monitored daily, and P0 refers to the day of litter discovery.
Wild animals	No wild animals were used in this study.
Reporting on sex	Dissociated cultures were prepared from pups of both sexes. There is no reported or discernible differences between sexes in electrophysiological properties of culture prepared at age P0-1.
Field-collected samples	No field collected samples were used in this study.
Ethics oversight	All procedures were carried out under PPL 70/8135 in accordance with UK Home Office regulations. Experiments conducted in the UK are licensed under the UK Animals (Scientific Procedures) Act of 1986 following local ethical approval.

Note that full information on the approval of the study protocol must also be provided in the manuscript.
