# Supplementary figures and images for: Proton-triggered rearrangement of the AMPA receptor N-terminal domains impacts receptor kinetics and synaptic localization
Source: Nat Struct Mol Biol. 2024 Aug 13;31(10):1601–13. doi: 10.1038/s41594-024-01369-5 (PMC11479944; doi:10.1038/s41594-024-01369-5)

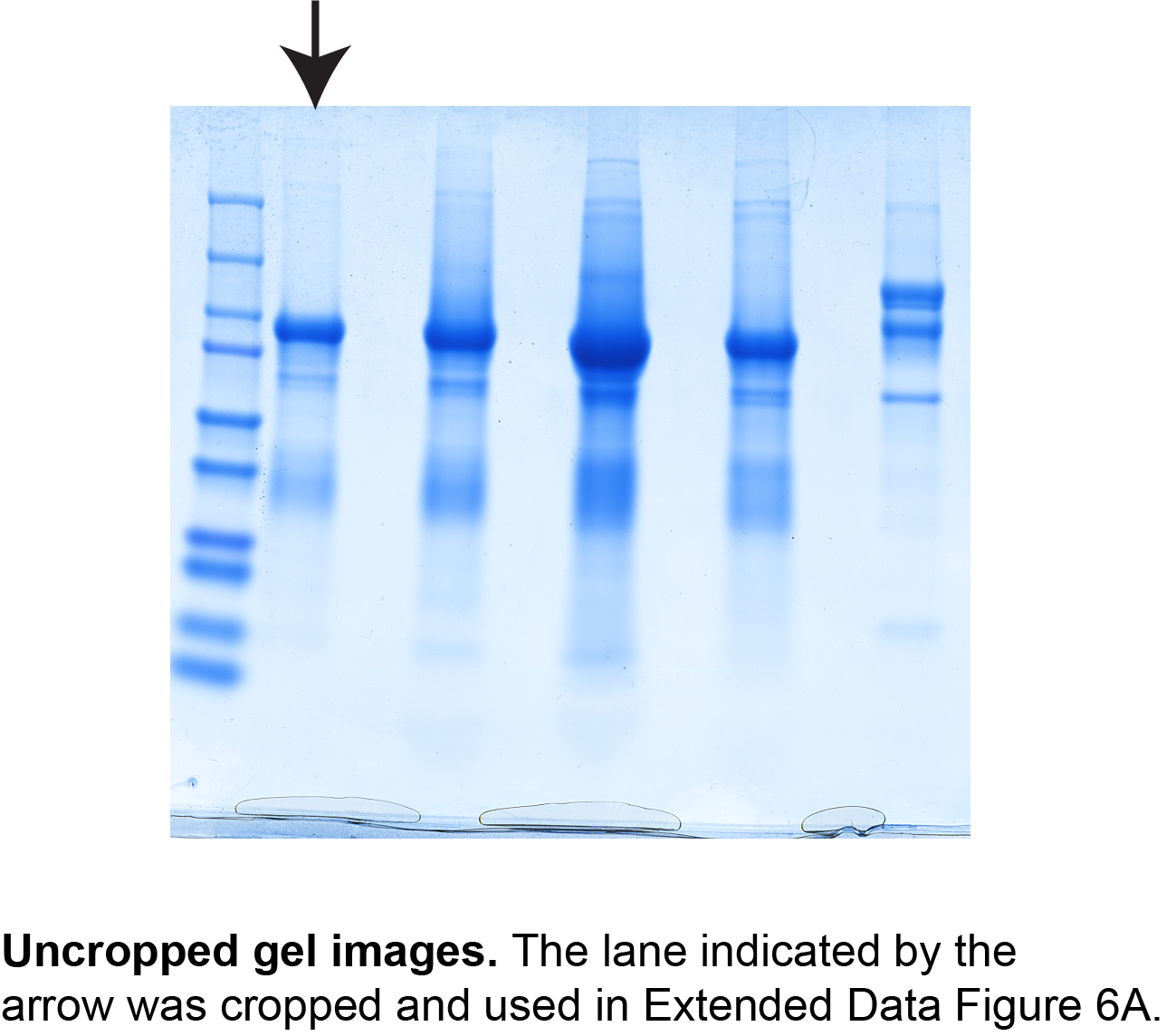

Supplement: Supplementary file 12 — Uncropped gel image of Extended Data Fig. 6b. [file 41594_2024_1369_MOESM12_ESM.tif]
